# Population Pharmacokinetics and Dosing Regimen Optimization of Meropenem in Cerebrospinal Fluid and Plasma in Patients with Meningitis after Neurosurgery

**DOI:** 10.1128/AAC.00997-16

**Published:** 2016-10-21

**Authors:** Cheng Lu, Yuyi Zhang, Mingyu Chen, Ping Zhong, Yuancheng Chen, Jicheng Yu, Xiaojie Wu, Jufang Wu, Jing Zhang

**Affiliations:** a Institute of Antibiotics, Huashan Hospital Affiliated to Fudan University, Shanghai, China; b Key Laboratory of Clinical Pharmacology of Antibiotics, Ministry of Health, Shanghai, China; c Department of Neurosurgery, Huashan Hospital Affiliated to Fudan University, Shanghai, China

## Abstract

Meropenem is used to manage postneurosurgical meningitis, but its population pharmacokinetics (PPK) in plasma and cerebrospinal fluid (CSF) in this patient group are not well-known. Our aims were to (i) characterize meropenem PPK in plasma and CSF and (ii) recommend favorable dosing regimens in postneurosurgical meningitis patients. Eighty-two patients were enrolled to receive meropenem infusions of 2 g every 8 h (q8h), 1 g q8h, or 1 g q6h for at least 3 days. Serial blood and CSF samples were collected, and concentrations were determined and analyzed via population modeling. Probabilities of target attainment (PTA) were predicted via Monte Carlo simulations, using the target of unbound meropenem concentrations above the MICs for at least 40% of dosing intervals in plasma and at least of 50% or 100% of dosing intervals in CSF. A two-compartment model plus another CSF compartment best described the data. The central, intercentral/peripheral, and intercentral/CSF compartment clearances were 22.2 liters/h, 1.79 liters/h, and 0.01 liter/h, respectively. Distribution volumes of the central and peripheral compartments were 17.9 liters and 3.84 liters, respectively. The CSF compartment volume was fixed at 0.13 liter, with its clearance calculated by the observed drainage amount. The multiplier for the transfer from the central to the CSF compartment was 0.172. Simulation results show that the PTAs increase as infusion is prolonged and as the daily CSF drainage volume decreases. A 4-hour infusion of 2 g q8h with CSF drainage of less than 150 ml/day, which provides a PTA of >90% for MICs of ≤8 mg/liter in blood and of ≤0.5 mg/liter or 0.25 mg/liter in CSF, is recommended. (This study has been registered at ClinicalTrials.gov under identifier NCT02506686.)

## INTRODUCTION

Postneurosurgical meningitis is a serious complication after neurosurgical operation, with an incidence rate of about 3% (1, 2) or even up to 20% if catheters are placed for drainage (3). It is associated with a high mortality rate and severe neurological sequelae if not identified at an early stage and treated in a timely manner with effective antimicrobial agents (4, 5). Gram-negative bacteria, especially antibiotic-resistant Acinetobacter baumannii, are emerging as important pathogens of postneurosurgical meningitis in recent years, though Staphylococcus aureus and coagulase-negative Staphylococcus are still more common (2, 3, 6, 7). The data on postoperative hospital-acquired bacterial meningitis from 2000 to 2009 in Huashan Hospital revealed that Gram-negative bacteria, primarily Acinetobacter baumannii, Klebsiella pneumoniae, and Pseudomonas aeruginosa, accounted for 42.1% of the pathogens isolated from postneurosurgical meningitis patients (8).

Meropenem is a potent broad-spectrum carbapenem with high levels of activity against Gram-negative bacilli, Gram-positive cocci, and anaerobic bacteria. It was recommended by the guidelines of both the Infectious Diseases Society of America in 2004 and the European Federation of Neurological Societies in 2008 (which are still effective) as one of the empirical treatments for bacterial meningitis (9, 10). In common with other β-lactams, it exhibits primarily time-dependent antimicrobial activity, and the pharmacokinetics/pharmacodynamics (PK/PD) index that best predicts clinical efficacy is the exposure time during which the drug concentration remains above MIC (*T*_>MIC_) for the pathogen (11, 12). There are a few pharmacokinetic and pharmacodynamic studies of meropenem in patients with bacterial meningitis (13–18); however, the patient numbers in these studies are usually limited. There are few population PK studies to describe meropenem population PK profiles for penetration across the blood-cerebrospinal fluid (CSF) barrier, and there is no PK/PD study in postneurosurgical meningitis patients to guide meropenem dosing regimen optimization for this specific patient group.

CSF drainage is a common operation in postneurosurgery patients to drain inflammatory mediator residual cells during the operation and to manage central nervous system (CNS) symptoms (19–26); however, it also results in drug loss. There is no PK/PD study in this patient group to recommend the proper CSF drainage rate to seek a balance between bacterial killing and postsurgery symptom management.

The aims of this study were (i) to develop a population PK model for meropenem after intravenous administration in postneurosurgical meningitis patients and (ii) to recommend clinical dosing regimen optimization for this patient group, including dose, dosing interval, infusion duration, and CSF drainage rate, via PK/PD evaluation.

(Part of this study was presented at ASM Microbe, 16 to 20 June 2016, Boston, MA, USA.)

## MATERIALS AND METHODS

### Study design.

This was a single-center, parallel, open-label, prospective clinical study conducted in Huashan Hospital Affiliated to Fudan University. It was approved by the Hospital Institutional Review Board of Huashan Hospital before execution and has been registered at ClinicalTrials.gov under identifier NCT02506686.

### Subjects.

Patients were included if inclusion and exclusion criteria were all satisfied. The inclusion criteria were as follows: (i) signed informed consent form provided; (ii) postneurosurgery patients with clinical diagnosis of probable bacterial meningitis (temperature of >37.5°C, signs of meningeal irritation [including neck rigidity, Kernig sign, and Brudzinski sign], and white blood cells in CSF at >300 × 10^6^/liter) or proven bacterial meningitis (if Gram-negative bacteria were identified from CSF culture); (iii) CSF samples could be taken; and (iv) at least 18 years of age. The exclusion criteria were as follows: (i) patients who did not receive at least 3 days of meropenem treatment or were hypersensitive to meropenem; (ii) patients who were receiving hemodialysis, had unstable vital signs, or had lumbar puncture contraindications and so were inappropriate for sample collection; (iii) patients with decompensated liver disease (e.g., Child-Pugh class B or C clinical classification or clinical evidence such as ascites or varices) or severe renal dysfunction (defined as creatinine clearance [CL_CR_] of ≤10 ml/min), status epilepticus, potential neurodegenerative diseases, and other meropenem contraindications, and (iv) pregnant (with a positive urine pregnancy test at screening) or lactating women.

### Dosing regimens.

The meropenem (0.5 g/vial) used in this study was manufactured by Dainippon Sumitomo Pharma. Meropenem at 1 g and 2 g was dissolved in 100 ml and 250 ml 0.9% sodium chloride solution, respectively, for injection. The infusion rate was kept at 1 g/h with the actual infusion start and end times recorded. The meropenem dosing regimen was determined according to the patient's body temperature, severity of systemic infection, CSF cell count, and pathogen. The total daily dose of meropenem was 3 to 6 g divided into 3 or 4 intravenous infusions. In most cases, meropenem at 1 g every 8 h (q8h) was given to patients with a white blood cell count of <1,000 × 10^6^/liter in CSF. Meropenem at 1 g q6h or 2 g q8h was given to patients with a white blood cell count of >1,000 × 10^6^/liter in CSF, depending on the patient's body temperature and general condition. The whole treatment lasted 3 to 14 days. Adverse events were observed and recorded per ICH good clinical practices (GCP) and China GCP. One predose CSF sample was collected and sent to the clinical microbiology department of the hospital for pathogen culture, isolation, identification, and antibiotic susceptibility testing. Treatment regimens were adjusted according to susceptibility if necessary.

### Blood and CSF sampling.

Blood and CSF samples were collected simultaneously after the fourth dose of meropenem for concentration testing. To increase the sample coverage of the concentration-versus-time curve, patients were randomly assigned to two groups for sample collection at predefined time points: during intravenous infusion and 10 min, 2 h, and 4 h after the end of infusion in group 1 and immediately at the end of infusion, 1 h and 3 h after the end of infusion, and immediately before administration of the next dose in group 2. Blood samples were collected from a vein of the arm without intravenous infusion. CSF samples were collected through lumbar cistern drainage or external ventricular drainage.

### Meropenem assay.

Plasma samples were separated by centrifugation and stored in duplicate along with CSF samples at −80 ± 10°C for future analysis. Validated high-pressure liquid chromatography (HPLC)-UV chromatographic separation methods were used to extract meropenem and detect its concentrations in plasma and CSF. Methodology details can be found in our previous publications (27, 28).

### Population PK model development.

A nonlinear mixed-effects model analysis was conducted using NONMEM version 7.3 (Icon Development Solutions, Ellicott City, MD) with the G77 FORTRAN compiler. The first-order conditional estimation method with interaction (FOCE-I) was used throughout the model-building process. One specific CSF compartment was added in the models to describe the meropenem disposition process in CSF (29–32), which made the model a hybrid semiphysiologic model. Models were selected based on several criteria, such as diagnostic scatter plots, an objective function value (OFV) decrease of 3.84 (α = 0.05, df = 1) for nested models, and the Akaike information criterion for nonnested models. The residual-based model diagnostic was performed using conditional weighted residuals. The covariate model building was performed in a stepwise fashion with forward inclusion and backward deletion. Variables screened as covariates were age, sex, body weight, body mass index (BMI), serum creatinine, creatinine clearance (CL_CR_), serum alanine aminotransferase (ALT), CSF white blood cell count, CSF red blood cell count, CSF absolute neutrophil count, CSF glucose, CSF protein, CSF chlorine, and concomitant medications (mannitol, dexamethasone, vancomycin, fosfomycin, nimodipine, and sodium valproate). CL_CR_ was estimated from the Cockcroft-Gault equation (33) using the age, body weight, and serum creatinine level of each subject. The covariate screening process was performed using visual (parameter-versus-variable scatter plots) and numerical (generalized additive modeling implemented in Xpose [v. 4]) approaches. Variables that passed the screening procedures were included in the model and tested for significance as a covariate based on the aforementioned model selection criteria. At the backward elimination step, covariates that did not increase the minimized OFV more than 6.63 (α = 0.01, df = 1) were eliminated from the final model.

### Bootstrapping and VPCs.

The bootstrap resampling method was used to evaluate the stability and robustness of the final PK model. Resampling with replacement generated 1,000 bootstrap data sets, and the final population PK model was fitted repeatedly to each of them. Ninety-five percent confidence intervals (CIs) for the final parameters were obtained from the bootstrap empirical posterior distribution. Visual predictive checks (VPCs) were performed by overlaying observed data points with 5th, 50th, and 95th percentile curves of 1,000 data sets simulated from the final model.

### Monte Carlo simulations.

The steady state of the concentration-time curve for meropenem on the basis of the model developed for the currently used dosage regimens (1 g q8h, 1 g q6h, and 2 g q8h at different infusion durations of 0.5 h, 1 h, 2 h, 3 h, 4 h, and continuous infusion) were obtained from 1,000 simulated virtual postneurosurgical meningitis patients with different CSF rate amounts per clinical practice. The CSF drainage rate was set at 0, 50 ml, 150 ml, and 250 ml per day.

### PK/PD analysis.

The free meropenem concentration in blood was corrected by a protein binding rate of 2% (34), while it was assumed that all meropenem in CSF is free. The fractions of patients achieving the PK/PD targets of 40% (plasma) and 50%/100% (CSF) *fT*_>MIC_ were calculated to approximate the probability of target attainment (PTA) (18). Target MICs for bacteria were selected according to most recent CLSI and EUCAST guidance and extended to higher MICs to seek a chance to manage more resistant bacteria. The PK/PD target was defined as the highest MIC with a probability of target attainment of at least 90%.

## RESULTS

### Patient characteristics.

A total of 82 patients (50 males and 32 females) with postneurosurgery meningitis were enrolled in this study, and initially 42 of them were treated with the dosing regimen of 1 g q8h, 19 with the regimen of 1 g q8h, and 21 with the regimen of 2 g q8h, according to their baseline conditions. They had different original diseases, including brain injury, brain bleeding, brain glioma, astrocytoma, acoustic neurinoma, trigeminal neurinoma, neurocytoma, meningioma, thalamic cyst, hemangioblastoma, encephalic angioma, cholesteatoma, etc. The baseline characteristics of all enrolled patients are shown in Table 1. All subjects signed informed consent forms by themselves. Forty patients were assigned to group 1 and 42 were assigned to group 2, according to the times of their blood and CSF sample collection.

**TABLE 1:** Demographic and baseline clinical data for patients

Parameter	Value (*n* = 82)
Sex, male/female	50/32
Age, yr (mean ± SD, range)	43.4 ± 13.1, 19–77
Height, cm (mean ± SD, range)	167.7 ± 7.2, 150–180
Weight, kg (mean ± SD, range)	65.2 ± 11.6, 41.5–100
BMI, kg/m^2^ (mean ± SD, range)	23.1 ± 3.5, 13.7–32.7
CL_CR_, ml/min (mean ± SD, range)	142.6 ± 52.8, 57.3–355.7
Body temp, °C (mean ± SD, range)	38.9 ± 0.6, 36.5–40.9
White blood cell count in CSF, 10^6^/liter (mean ± SD, range)	2,139.5 ± 2,877.7, 1–20,000
White blood cell count in blood test, 10^9^/liter (mean ± SD, range)	12.6 ± 4.4, 4.2–23.9
Proteins in CSF, g/liter (mean ± SD, range)	1.9 ± 1.5, 0.2–8.7
Glucose in CSF, mmol/liter (mean ± SD, range)	2.4 ± 1.6, 0.3–7.9
Underlying disease, no.	
Tumor	65
Trauma	8
Other	9
Combination treatment, no.	
Vancomycin/norvancomycin	59
Other	7
CSF daily drainage vol, ml (mean ± SD, range)	126 ± 81, 0–350

### Population PK modeling.

**FIG 1:**
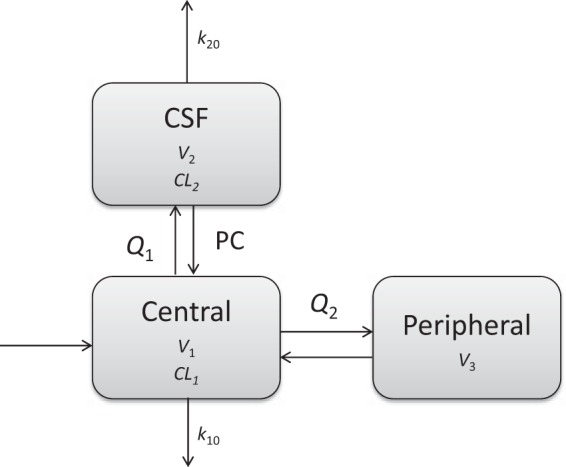
Pharmacokinetic structural model for meropenem. *V*_1_, apparent volume of central compartment; CL_1_, clearance of central compartment; *V*_2_, volume of CSF compartment; CL_2_, clearance of CSF compartment; *Q*_1_, intercompartmental clearance between central and CSF compartments; *Q*_2_, intercompartmental clearance between central and peripheral compartments; PC, transfer multiplier between central and CSF compartments; *V*_3_, volume of peripheral compartment; *k*_10_, elimination rate constant for central compartment; *k*_20_, elimination rate constant for CSF compartment.

A total of 315 plasma and 297 CSF meropenem concentration data points were included in developing the population PK model. The final model contained one central compartment, one peripheral compartment, and one CSF compartment, as shown in Fig. 1. The initial estimation of population CSF volume was set at 0.15 liter (35), with the upper and lower limits from 0.13 to 0.17 liter to reflect potential difference. During the model-building process, 0.13 liter fit the data best, so the CSF compartment volume was fixed to 0.13 liter. PC is defined as transfer multiplier between the central and CSF compartments. The intercompartmental rate constants were defined as *K*_12_ = *Q*_1_ × PC/*V*_1_, *K*_21_ = *Q*_1_/*V*_2_, and *k*_20_ = daily CSF drainage volume/24.

**FIG 2:**
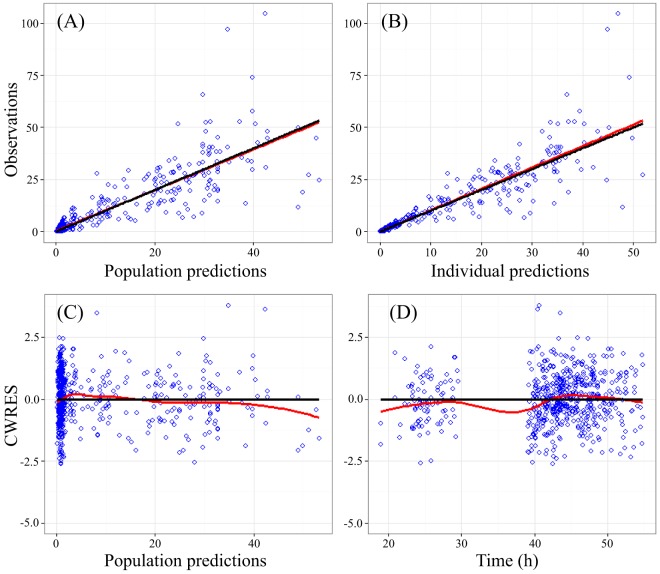
Goodness-of-fit plots for the final population PK model. (A) Plot of observed meropenem concentrations versus population predictions. Black line, line of identity; red line, data smoother. (B) Plot of observations versus individual predictions. Black line, line of identity; red line, data smoother. (C) Plot of population weighted residuals (CWRES) versus population predictions. Black line, zero-slope line; red line, data smoother. (D) Plot of conditional weighted residuals versus time. Black line, zero-slope line; red line, data smoother. Predicted concentrations are in milligrams per liter; time is in hours.

**FIG 3:**
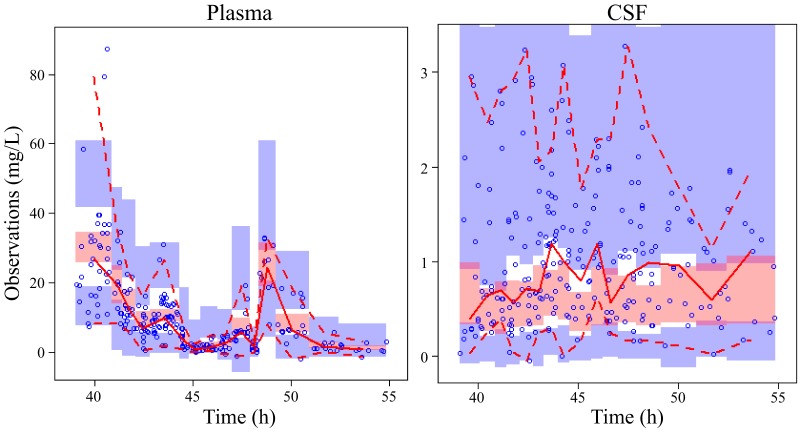
Visual predictive check plots for the drug concentration in plasma (left) and CSF (right). Open circles represent observed concentrations. The red solid line represents the median of the observations. The red dashed lines represent the 5th and 95th percentiles of the observations. The shaded areas represent the simulation-based 95% confidence intervals for the fifth (blue shaded at the bottom), median (red shaded), and 95th (blue shaded at the top) percentiles of the predicted data.

The estimated population pharmacokinetics (PPK) parameters are shown in Table 2. No covariate was identified to have significant impact on the model from covariate screening. Goodness-of-fit plots for the final model were evaluated and showed no apparent visual bias for the predictions, as shown in Fig. 2. The 95% confidence intervals for the parameters from the final model, from 1,000 bootstrap runs, are presented in Table 2, and the VPC plots are shown in Fig. 3. All parameter estimates were within the ranges of the 95% confidence intervals from 1,000 bootstrap runs, indicating the robustness of the final model. The visual predictive check confirmed the predictive performance of the model. There was a resemblance between observed and simulated data. Nearly all the observations were within the percentile range. The very few observations which were outside the percentile range were randomly scattered and not aggregated at a particular time point. The eta shrinkage range of the parameters was 7.75% to 69.7%, and the epsilon shrinkage was 12.2%. These findings imply that the final model had adequate predictive ability to describe the measured meropenem concentrations.

**TABLE 2:** Pharmacokinetic parameter estimates and bootstrap confidence intervals of the final model

Parameter (units)^*a*^	Estimate		Between-subject variability (%)	
	Mean	Confidence range, 2.5th–97.5th percentile (bootstrap)	Mean	Confidence range, 2.5th–97.5th percentile (bootstrap)
*V*_1_ (liters)	17.9	16.1–19.5	13.2	0–20.0
*V*_2_ (liters)	0.13		37.4	0–71.4
CL_1_ (liters/h)	22.2	20.5–24.0	22.4	17.3–26.5
*Q*_1_ (1/h)	0.010	0.010–0.010	84.4	50.0–104
PC	0.172	0.140–0.220	39.4	20.0–48.0
*V*_3_ (liters)	3.84	3.04–4.95		
*Q*_2_ (1/h)	1.79	1.21–2.99		
Residual error (%)	34.9	31.6–38.7		

a *V*_1_, apparent volume of central compartment; CL_1_, clearance of central compartment; *V*_2_, volume of CSF compartment; CL_2_, clearance of CSF compartment; *Q*_1_, intercompartmental clearance between central and CSF compartments; *Q*_2_, intercompartmental clearance between central and peripheral compartments; PC, transfer multiplier between central and CSF compartments; *V*_3_, volume of peripheral compartment.

### Monte Carlo simulation and PK/PD analysis.

**FIG 4:**
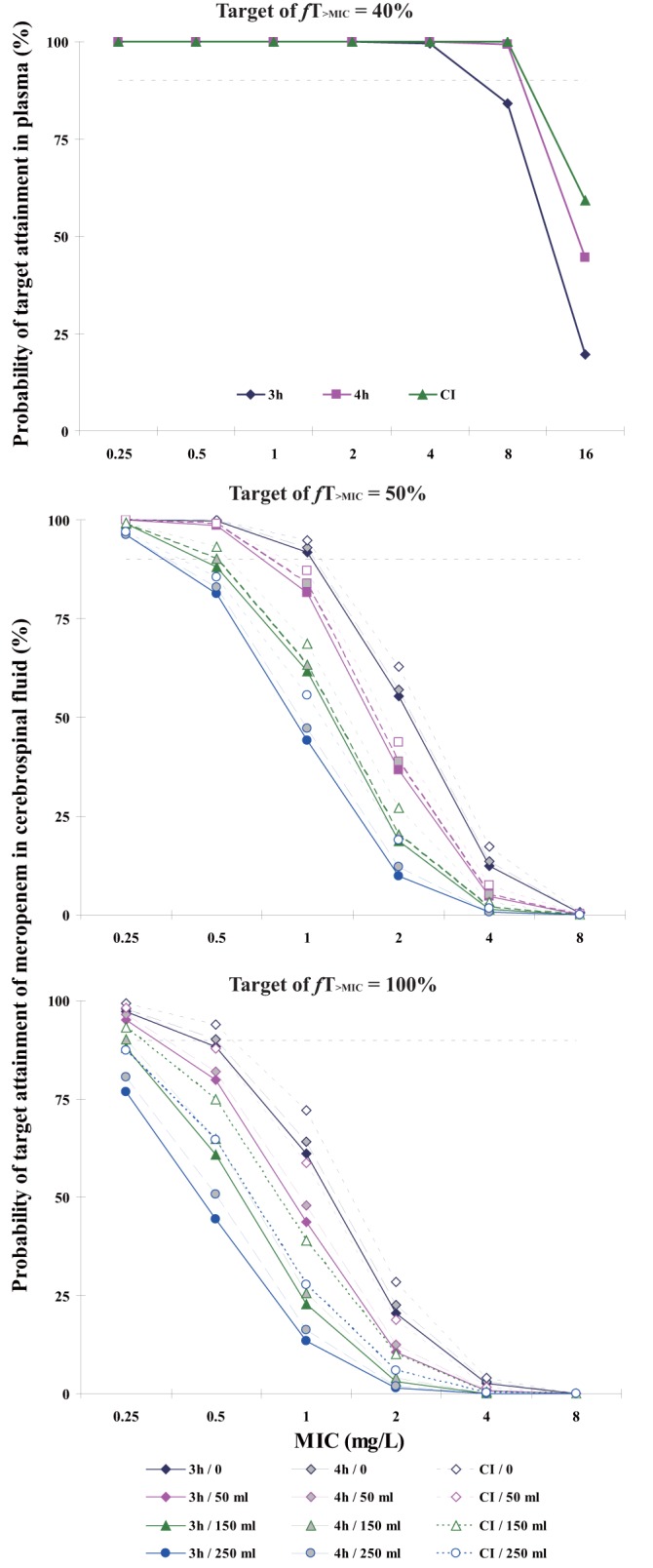
Probabilities of target attainment of meropenem regimens of 2 g q8h. (Top) Plasma, with a PK/PD target of 40% *fT*_>MIC_. The PTAs do not change at different CSF drainage rates. The infusion duration of each dose is indicated. CI, continuous infusion. (Middle and bottom) CSF, with a PK/PD target of 50% *fT*_>MIC_ (middle) or 100% *fT*_>MIC_ (bottom). The infusion duration of each dose (hours) and the CSF drainage rate (milliliters per day) are indicated.

The trends we saw from Monte Carlo simulation results are that the probability of target attainment (PTA) increases as the total daily dose increase, while for the same dose, the longer the infusion time, the higher the PTA. CSF drainage has no impact on PTAs in plasma, but the more that CSF drained every day, the lower the PTA was in CSF. Under all circumstances of regimens of 1 g q8h and 1 g q6h including no CSF drainage, the probabilities of achieving 50% and 100% *T*_>MIC_ in CSF are less than 90% for MICs of >0.25 mg/liter and >0.5 mg/liter, respectively, so these regimens are not recommended. The PTAs of regimens of 2 g q8h are shown in Fig. 4. Although meropenem could be administered as a continuous infusion (36) and continuous infusion shows the highest PTAs in the simulation results, its short room temperature stability of ∼4 to 6 h makes these regimens less attractive (37). A 4-h infusion with a limited CSF drainage rate (less than 150 ml/day) is recommended from the simulations. In plasma, this has a >90% probability of achieving 40% *T*_>MIC_ for MICs of ≤8 mg/liter. In CSF, it has a >90% probability of achieving of achieving 50% and 100% *T*_>MIC_ for MICs of ≤0.5 mg/liter and ≤0.25 mg/liter, respectively, and has a >80% probability of achieving 50% and 100% *T*_>MIC_ for MICs of ≤1 mg/liter and ≤0.5 mg/liter, respectively.

## DISCUSSION

Postneurosurgery bacterial meningitis remains a severe disease associated with high mortality rates. Although CSF culture is the “gold standard” for diagnosis of meningitis and is important to establish the susceptibility of the causative microorganism to rationalize treatment, it is time-consuming. Rapid initiation of effective empirical treatment is still crucial for patient outcomes (38–43). There were some studies on prolonged infusion of meropenem; however, there was only one population PK study (18) and two case studies (44, 45) focused on using meropenem to treat nosocomial CNS infection. The population PK study enrolled only 10 patients in the meropenem group, and the population apparent CSF volume was estimated by their model to be 82.44 ± 23.79 liters. Major limitations of their study were that (i) they did not consider drug loss by CSF drainage and (ii) they investigated only an infusion duration of 0.5 h. To the best of our knowledge, our study included the largest number of patients, is the first study which introduced CSF drainage as an element of the dosing regimen, and is the first study which has recommended a dose, dosing interval, infusion duration, and CSF drainage rate together for postneurosurgery patients.

In plasma, the CSF drainage rate had no impact on probabilities of target attainment. This may be because the target was set at 40% and the amount of drug elimination from CSF drainage was limited compared to the total drug amount in plasma. In CSF, the CSF drainage rate had a significant impact on the drug amount in CSF, though the trade-off between postsurgery symptom management and maintaining drug concentrations in infection site should be carefully made.

The final model is more than a classic population PK model because it introduced a CSF compartment. The fixed CSF compartment volume was a little lower than the reported physiological volume (35), which might be because Chinese people have lower body weight and smaller body size and so might have lower CSF volume than western people. There was no significant covariate identified, which might be due to the relative sparseness of the individual sampling schedule. All subjects we included had normal or mildly impaired renal function, and that might be the reason that CR_CL_ was not identified as a covariate. The variance of PK parameters was relatively high but still meaningful, and this might be because the patients had different baseline disease conditions, different surgical operations, and different demographic characteristics. Our approach can also be used to investigate and recommend favorable dosing regimens for other antibiotics to manage this indication.

The PK/PD target for bactericidal activity in CSF is still a subject of debate; while some investigators choose 50% and 100% *fT*_>MIC_ (18, 46), some think that although not ideal, having bacteriostatic targets of 20 to 30% *fT*_>MIC_ plus white blood cells in CSF might be sufficient to eradicate the organism (44, 45). As CNS infection is considered severe, we still believe that 50% and 100% *fT*_>MIC_ should be used.

A second debate is whether CSF drug concentration is a good surrogate of infection site concentration. Ethically, the collection of CSF samples does not require additional operations for the patients and hence does not bring additional risks. Although there was a study which measured the meropenem concentration in cerebral extracellular fluid (ECF) in two patients via brain microdialysis (47), this approach can be used only in patients with brain injury.

A third debate is what the most appropriate CSF drainage rate is. Although 50 ml/day provides a higher PTA for bacterial killing, it might not be enough to manage the postsurgery syndromes in some patients. Therefore, less than 150 ml/day was recommended, and the clinicians could be reminded to drain as little CSF as possible as long as the syndromes could be well managed.

Our study has some limitations. First, it was not designed to assess clinical outcome; therefore, no control was included. Second, only 8 out of 82 enrolled patients had a positive result from CSF bacterial culture, which indicated that the diagnosis criteria for probable meningitis need to be improved. The bacteria isolated included Acinetobacter baumannii (*n* = 6, with meropenem MICs of 0.25, 0.5, 0.5, 16, 32, and 32 mg/liter), Klebsiella pneumoniae (*n* = 1, with a meropenem MIC of 1 mg/liter), and Stenotrophomonas maltophilia (*n* = 1, with a meropenem MIC of 4 mg/liter). The 3 patients who were infected by meropenem-resistant bacteria (MICs of 32, 32, and 4 mg/liter) were withdrawn from the study and switched to the other treatment regimens, and the clinical outcomes were not recorded. It is worthy of mention that the individual fitting plots for the 8 subjects who had pathogens isolated from CSF imply that the final model predicted the concentrations in this patient population well also.

In summary, our current study of population PK and PK/PD in postneurosurgery meningitis patients found that 2 g of meropenem every 8 h, administered as a 4-hour infusion with a limited CSF drainage rate (less than 150 ml/day), may provide the highest possibility of target attainment. However, further large, well-defined clinical trials with this patient population are required to confirm these findings.
